# Draft Genome Sequence of Cronobacter sakazakii H05, Sequence Type 156, Isolated from a Spiced Ground Beef Dish, Kitfo

**DOI:** 10.1128/mra.00643-22

**Published:** 2022-10-13

**Authors:** Behailu B. Eshetea, Junia Jean-Gilles Beaubrun, Abdullah Mashraqi, Broderick Eribo

**Affiliations:** a Howard University, Department of Biology, Washington, DC, USA; b Biological Analysis, Division Public Health Command Europe Laboratory Science, Kirchberg Kaserne, Landstuhl, Germany; c Jazan University, Biology Department, College of Science, Jazan, Saudi Arabia; d Howard University, Department of Biology, Washington, DC, USA; University of Maryland School of Medicine

## Abstract

Cronobacter sakazakii is a foodborne pathogen that causes severe illness in neonates and the elderly. Here, we present the genome sequence of C. sakazakii H05 sequence type 156 (ST156), CC21 strain from Kitfo, resulting in a genome size of 4,495,386 bp, with 4,340 coding sequences and a G+C content of 56.85% after assembly and annotation.

## ANNOUNCEMENT

*Cronobacter* is a Gram-negative opportunistic pathogen found widespread in the environment; it is an important foodborne pathogen that can infect humans and cause life-threatening infections, such as meningitis, septicemia, and necrotizing enterocolitis (1). It is a high risk to infants fed contaminated formula with a fatality rate of ~27% (2) and has attracted public attention (3). Its recovery from various low-moisture foods, such as infant formula, including cereals, nuts, peanut and nut butter, chocolate, spices, and fresh vegetables, has been documented (4–7). In addition, confirmed *Cronobacter* isolates from a ground beef-based ready-to-eat dish called Kitfo (8) was sequenced and analyzed to expand our knowledge about these pathogens.

The Kitfo samples enriched in tryptic soy broth overnight at 32°C were plated into R&F (Enterobacter sakazakii) chromogenic plating medium at 32°C for 24 h (9). The isolate with a blue-black presumptive colony morphology was purified several times by plating a single colony. Finally, the isolate was confirmed using a Vitek 2 system (bioMérieux) (10). Subsequently, the overnight culture kept at 32°C in tryptic soy broth was used for DNA extraction using the Wizard genomic DNA kit (Promega, Madison, WI). Whole-genome sequencing (WGS) libraries were constructed using the Nextera XT DNA sample preparation kit (Illumina, San Diego, CA). The bacterial genome was sequenced using the MiSeq platform (Illumina) using a 250-bp paired-end read v2 kit. The identification was further verified using WGS. All bioinformatics analyses were performed using the Pathosystems Resource Integration Center (PATRIC) v3.6.12 platform (11). FastQC v0.11.7 (12) confirmed that the fastq files were good quality with 1,649,960 total reads. The PATRIC Comprehensive Genome Analysis Service used SPAdes for the *de novo* assembly of the reads (13). Default parameters were used for all software unless otherwise specified.

The assembly produced 39 contigs with coverage of 183× and *L*_50_ and *N*_50_ values of 4 and 481,817 bp, respectively. The draft genome of C. sakazakii H05 revealed 4,495,386 bp with an average G+C content of 56.85%. Genome annotation was performed with NCBI Prokaryotic Genome Annotation Pipeline (PGAP) at the National Center for Biotechnology Information (NCBI). The genome had a total of 4,175 protein-coding sequences (CDS), 74 tRNA gene sequences, 3 rRNA genes, and 123 CRISPR direct repeat arrays with 119 spacers. The genome annotation showed the presence of efflux pumps, such as AcrAB-TolC, AcrAD-TolC, and AcrEF-TolC. It also contained antibiotic resistance genes, such as *FosA*, *marA*, *marB*, *MacB*, *MarR*, *gyrA*, and *gyrB*. Moreover, the strain harbors an IncFIB(pCTU3)-like plasmid. The multilocus sequence type (MLST) for H05 is sequence type 156 (ST156), CC21, based on the PubMLST database (http://pubmlst.org/cronobacter).

### Data availability.

The genome sequence of C. sakazakii H05 was submitted to NCBI GenBank under BioProject number PRJNA688536. The sequence is deposited under accession number JAEOAX000000000. The version described in this paper is the first version, JAEOAX000000000. The Sequence Read Archive (SRA) submission is available under accession number SRR21616149.
